# Assertiveness in Nursing: A Systematic Review of Its Role and Impact in Healthcare Settings

**DOI:** 10.3390/nursrep15030102

**Published:** 2025-03-15

**Authors:** Maha R. Al-hawaiti, Loujain Sharif, Hala Elsayes

**Affiliations:** 1 Psychiatric and Mental Health Nursing Department, Faculty of Nursing, King Abdulaziz University, Jeddah 21551, Saudi Arabia; lsharif@kau.edu.sa (L.S.); halsayes@kau.edu.sa (H.E.); 2 Nursing Administration Services, Duba General Hospital, Ministry of Health (MOH), Tabuk Health Cluster, Tabuk 47717, Saudi Arabia; 3 Psychiatric and Mental Health Nursing Department, Faculty of Nursing, Tanta University, Tanta 31511, Egypt

**Keywords:** assertiveness, nursing, communication, patient care, interprofessional relationships

## Abstract

**Background:** Assertiveness in nursing is crucial to improving communication, reducing interpersonal tensions, and improving healthcare outcomes. **Objective:** The objective of this systematic review was to identify and synthesize the literature on assertiveness in nursing, focusing on nurses’ perspectives regarding its role, influencing factors, challenges, and impact within complex healthcare settings. **Methods:** This review followed the Preferred Reporting Items for Systematic Reviews and Meta-Analysis (PRISMA) guidelines. An integrated mixed methods approach was used to capture the multifaceted nature of assertiveness in nursing. The evaluation covered both qualitative and quantitative investigations, concentrating on quantitative publications between 2018 and 2024 and qualitative publications without a time limit. Diverse methodologies were incorporated using the SPIDER framework. A comprehensive electronic search was conducted across six databases: PubMed, Medline, CINAHL, PsychInfo, Wiley Online Library, and Web of Science. Ultimately, 15 research papers were included in the review out of 9490 publications that were initially identified. Included studies were critically appraised using the Critical Appraisal Skills Program (CASP) and the Joanna Briggs Institute Critical Appraisal checklists. **Results:** A total of 9490 studies were identified, of which 15 (eight quantitative and seven qualitative) were included in this review. The review and subsequent analysis revealed five major themes: (1) facilitators of assertiveness; (2) barriers to assertiveness; (3) assertiveness training; (4) interaction with other staff; and (5) patient relationships. **Conclusions:** Assertiveness is essential for proficient nursing practice, especially in complex healthcare environments. It facilitates improved communication, alleviates stress, and augments patient care. Future research should explore the long-term benefits of assertiveness training and its influence across various cultural settings.

## 1. Introduction

In the field of nursing, being assertive is an important communication skill that is linked to teamwork, confidence, and overall patient care [1,2,3]. Omura et al. explain that assertiveness in nursing is the capacity to thoughtfully communicate concerns about matters that could affect patient safety and to discuss viewpoints with colleagues, including those in leadership positions [3]. Assertiveness is critical for organizational efficiency as it affects nurse–patient relationships, patient advocacy, safety protocols, negotiation abilities, and job satisfaction [1,2,4,5].

Building on the importance of assertiveness in nursing, it is particularly vital in complex healthcare settings, where effective communication directly influences patient outcomes and care quality. In nursing, assertiveness is viewed as a core communication skill that enhances patient care and contributes to competency in acute care and recovery [4]. Combining assertiveness with emotional intelligence empowers nurses to navigate the complexities of their field, build strong relationships, and provide optimal care [6].

Despite the recognized importance of assertiveness in nursing, there are notable gaps in the literature, particularly regarding nurses’ perspectives and the influence of cultural and situational factors. While some qualitative studies exist, their coverage is limited, necessitating further exploration. Filling these gaps is critical to ensure customized interventions and educational initiatives that address obstacles that could impede the advancement and utilization of assertive communication skills in complex healthcare facilities. In 2018, Omura et al. published a systematic review titled “The Effectiveness of Assertiveness Communication Training Programs for Healthcare Professionals and Students”, which primarily focused on quantitative outcomes [3]. While valuable, its findings highlighted the need for updated reviews to address recent advancements in assertiveness within nursing and to guide future interventions aimed at enhancing nursing practice. This review aims to systematically examine assertiveness in nursing by synthesizing the literature on nurses’ perspectives, influencing factors, challenges, and its impact in complex healthcare environments.

## 2. Materials and Methods

### 2.1. Design

A systematic review design was employed in line with the Preferred Reporting Items for Systematic Reviews and Meta-Analyses guidelines (PRISMA) [7]. This review adopted an integrated approach, synthesizing both qualitative and quantitative research to thoroughly explore assertiveness in nursing. This mixed methods approach was deliberately chosen to encapsulate the multifaceted nature of assertiveness in nursing, providing a holistic examination of its impacts [8]. By integrating qualitative insights with quantitative data, this approach facilitated a deeper understanding of nurses’ experiences and the effects of assertiveness on patient care. Mixed methods research enhances data richness by combining diverse perspectives, allowing for more rigorous and flexible data collection while amplifying the voices of under-represented populations [9,10]. The systematic review process was carried out in specific stages, as detailed in the following sections.

### 2.2. Eligibility Criteria

To ensure the inclusion of recent and relevant empirical findings, quantitative studies were limited to publications between 2018 and 2024. In contrast, qualitative studies were included without time restrictions to capture broader historical perspectives on assertiveness in nursing.

The review adhered to the SPIDER framework (Sample, Phenomenon of Interest, Design, Evaluation, Research type) (Table 1) to systematically identify studies exploring nurses’ assertiveness and its impact on patient care and interprofessional relationships. The inclusion criteria focused on primary research involving registered nurses in various healthcare settings, ensuring a comprehensive understanding of the role, impact, and influencing factors of assertiveness in nursing. Only English-language peer-reviewed studies were included.

Studies were excluded if the research did not directly assess nurses’ assertiveness, including studies centered solely on patients or other healthcare professionals. Additional exclusion criteria included the following: opinion pieces, study protocols, editorials, conference abstracts, case studies, conference proceedings, and theses/dissertations. These were excluded to maintain methodological rigor.

This systematic review builds upon a 2017 foundational review, which predominantly examined quantitative outcomes. The broader inclusion of qualitative studies in this review ensures a deeper exploration of historical trends and contextual influences on assertiveness [10]. This approach is particularly valuable in interpersonal domains like nursing, where qualitative research can offer rich insights into attitudes, behaviors, challenges, and contextual factors.

### 2.3. Electronic Databases

A comprehensive literature search was executed across six electronic databases: PubMed, Medline, CINAHL, PsychInfo, Wiley Online Library, and Web of Science.

### 2.4. Search Strategy

The search parameters were restricted to English-language publications. The search was limited to 2018 to 2024 for qualitative studies with no date restrictions included in the qualitative searches. The search terms applied were based on MESH terms: “nurses”, “nursing”, and “assertiveness”.

### 2.5. Study Selection

**Figure 1:**
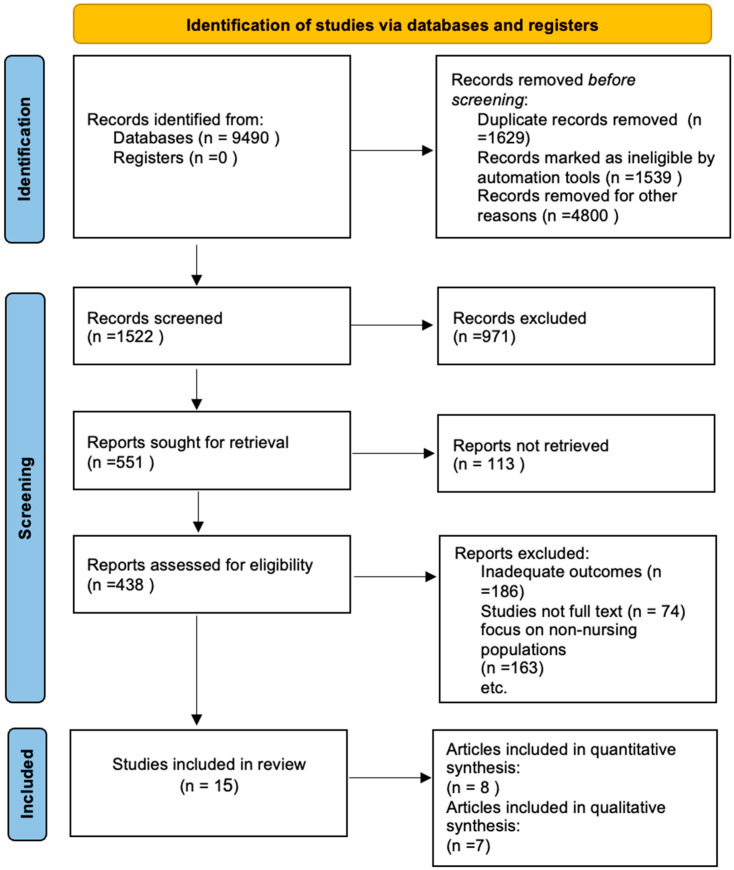
PRISMA flow diagram of the studies selection process (Page et al., 2020) [7].

Following the initial search, 9490 publications were filtered based on their titles and abstracts, resulting in 1522 articles for assessment. After further examination, 971 papers were excluded due to inadequate outcomes, mismatched demographics, irrelevant publication kinds, language limitations, and duplication. Fifteen publications were selected for inclusion: four used a quasi-experimental design, one used an experimental design, three used a cross-sectional design, and seven used qualitative methods. To ensure the utmost relevance, articles published over two decades prior to this review were omitted. The detailed literature search and study selection is demonstrated in the accompanying PRISMA flowchart (Figure 1). Firstly, author (M.R.A.-h.) assessed all titles for their possible relevance to the population and outcome. Secondly, two authors (L.S. and M.R.A.-h.) independently reviewed titles and abstracts to see if they met the inclusion criteria. Thirdly, two authors separately assessed the entire material, and, in the event of a discrepancy, a third author (H.E.) would review and settle the selection.

### 2.6. Data Extraction

The lead author (M.R.A.-h.) led the data extraction process using a structured chart that was created to collect essential elements from each article. The extracted data were reviewed independently by authors LS and HE to ensure accuracy and completeness. The data extraction table included fields such as author, year, country, study objective, sample characteristics, work environment, design, tools, results, and conclusions. This guaranteed a uniform and detailed retrieval of data, facilitating a comprehensive understanding of the scope and influence of assertiveness in nursing.

### 2.7. Quality Appraisal and Assessment of Risk of Bias

All references and abstracts were independently evaluated by two reviewers using predefined inclusion/exclusion criteria and assessed for quality using tools from the Critical Appraisal Skills Program (CASP) and the Joanna Briggs Institute (JBI). Discrepancies were resolved through consensus to minimize bias, ensuring the reliability of the process. The appraisal revealed that the seven qualitative studies synthesized scored 20 overall, while the five quasi-experimental studies, assessed with the JBI appraisal tool for risk of bias [11], scored between 20 and 27. The analytical cross-sectional studies, evaluated using the JBI Checklist for Analytical Cross-Sectional Studies [12], exhibited scores ranging from 17 to 22, indicating acceptable to good quality across all included studies (Table A1, Table A2 and Table A3).

### 2.8. Data Analysis

Given the heterogeneity in study designs and intervention content, a meta-analysis was not feasible. A narrative synthesis approach was adopted, which is commonly used in systematic reviews. This is a textual approach that analyzes the relationships within studies and between publications to synthesize the results. The key parameters taken into consideration during the analysis were methodologies and findings. This approach facilitated a comprehensive exploration of the effectiveness and impact of assertive interventions in nursing. The findings of this review are presented under five key themes: assertiveness training, barriers, facilitators, patient relationships, and interaction with other staff.

## 3. Results

### 3.1. Study Characteristics

As indicated in Table 2, the 15 studies reviewed were published between 2009 and 2023. The total sample size was 715 among the included quantitative studies. Of the quantitative studies, n = 4 used a quasi-experimental design, n = 1 an experimental design [1,13,14,15,16], and n = 3 a cross-sectional [17,18,19]. Though smaller in scale, the seven qualitative studies offered an in-depth exploration of assertiveness and communication dynamics among a combined total of 156 nurses, providing valuable qualitative perspectives on the topic (Table 2).

### 3.2. Analytical Findings

Five themes emerged from the findings of this review: (1) facilitators of assertiveness; (2) impediments to assertiveness; (3) training, (4) contact with other staff; and (5) the patient connection (Table 3). The prominent theme throughout the review was facilitators of assertiveness which appeared in eleven of the fifteen publications, followed by barriers to assertiveness and assertiveness training which emerged from eight publications. Contact with other staff appeared in seven publications, while patient relationships appeared in five publications.

#### 3.2.1. Facilitators of Assertiveness

The literature identifies key factors that promote assertiveness in nursing (Table 4). A significant factor is the work culture; nurses are more assertive in supportive environments and with colleague support [24,25]. Studies by Mansour and Mattukoyya and Lee et al. highlight that a supportive culture fosters safe spaces for assertive communication [20,22]. For instance, unit manager support enhances nurses’ willingness to voice concerns about patient safety [20,22]. Lee et al. also noted that a non-judgmental, non-punitive culture encourages nurses to speak up, especially when they are designated as safety champions [22].

The literature indicates that exploring personal values, feelings, and beliefs enhances nurses’ assertiveness [16,22]. Mahmoudirad et al. found that bravery, self-confidence, and religious beliefs contribute to assertiveness in nursing [23]. They noted that personal factors like “trust in God”, “working for God’s sake”, and “self-confidence” empower nurses to assert themselves. Law and Chan highlighted a nurse’s professionalism and patient-oriented actions as examples of assertiveness [24]. Additionally, prior positive assertive experiences can foster personal growth and encourage nurses to advocate for patient safety [24].

Nurses’ experience and knowledge enhance their credibility and confidence to assert themselves [25]. Wehabe et al. found that over three quarters of head nurses are highly assertive, attributed to their extensive experience, age, and qualifications [17]. Mohammed et al. similarly reported that older, more qualified nurses tend to be more assertive, highlighting a positive correlation between assertiveness, qualifications, and experience [15]. Conversely, Oducado and Montaño noted that nurses aged 26 and under with less than five years of experience were less assertive [18]. Additionally, Khanam et al. indicated that poor decision-making skills lower assertiveness [16]. Overall, the literature suggests that work experience significantly enhances assertiveness [17].

Three studies found that nurses’ responsibility for patient safety encouraged assertiveness [18,21,22]. Omura et al. noted that a few participants were motivated to provide person-centered care, which facilitated assertive communication [21]. Additionally, assertiveness training programs were identified as beneficial for enhancing assertiveness in nursing. Marahatta and Koirala emphasized the importance of hospital management in implementing educational programs, finding that training at least twice a year is necessary to improve nurses’ assertiveness [19].

#### 3.2.2. Barriers to Assertiveness in Nursing

A stigma around assertiveness persists in nursing. Omura et al. found that some nurses fear being seen as impolite when assertive [25]. Mansour and Mattukoyya noted that criticism can lead to negative self-perception, affecting assertiveness [20]. Additionally, Oducado and Montaño and Khanam et al. reported that nurses worry about reprimands and repercussions from management for assertive behavior, particularly concerning job performance reviews [16,18].

Healthcare hierarchies and power differentials hinder assertive communication among nurses, affecting their ability to make requests and communicate effectively [26]. Lee et al. and Omura et al. note that these structures often intimidate nurses, preventing them from speaking up around senior colleagues and physicians [22,24,25]. Oducado and Montaño found that Filipino nurses are less assertive with higher-ups. Additionally, harsh criticism can demoralize newly qualified nurses, leaving them feeling powerless [18,20]. An inadequate support system and lack of role models also negatively impact nurses’ assertiveness [16]. Heavy workloads further discourage nurses from addressing patient-related issues, as they fear added stress and blame, sometimes leading them to skip meals to manage their tasks. For instance, a nurse observed a resident ignoring infection control protocols but did not intervene while managing her workload. Overall, the literature highlights that these hierarchical barriers impede effective patient care and open dialogue.

Cultural norms significantly impact assertiveness. In Egypt, people often avoid saying no to please others, even at their own expense. In Japan, nurses prioritize group consensus and harmony, which restricts assertive communication [21]. They often view raising one’s voice as inappropriate and may feel conflicted about being assertive while maintaining professional respect. This emphasis on group harmony creates barriers to assertiveness, as noted by Lee et al., where nurses worry about being perceived as arrogant or disruptive [22]. Values of familiarity and group identification further deter assertiveness, especially in cultures prioritizing harmony, like Japan [25]. Nurses also find it challenging to assert themselves with unfamiliar individuals and often seek others’ opinions before speaking up, which can hinder their confidence to contribute in meetings.

#### 3.2.3. Assertiveness Training

Four of the fifteen studies indicated that training for nurses enhances self-confidence and self-actualization. Abdelaziz et al. [1] found that an assertiveness training program significantly improved assertiveness skills, psychological well-being, and work engagement in novice psychiatric nurses. This type of training helps individuals assert themselves without infringing on others’ rights [1,14]. Nemati et al. reported increased assertiveness and self-confidence among nurses following the training [13]. Similarly, Khanam et al. noted that assertiveness training for early career psychiatric nurses significantly boosted their assertiveness, psychological well-being, and job engagement [16]. Abdelaziz et al. also highlighted improvements in nurses’ psychological well-being and self-esteem, while Mostafa et al. found significant increases in self-esteem scores pre- and post-training [1,14]. Wahebe et al. noted that the training enhanced nurses’ ability to discuss decisions confidently and collaborate effectively [17]. Overall, assertiveness training can significantly improve assertiveness and self-confidence.

Abdelaziz et al. [1] emphasize the importance of teaching assertion skills to early career psychiatric nurses to foster professionalism. Similarly, Mohammed et al. found that training significantly improved head nurses’ professional knowledge [15]. Mastering assertion skills through training is vital for nurses at all career stages, as it enhances professionalism and promotes effective communication with colleagues, superiors, and patients [13]. Mostafa et al. noted a significant increase in communication skills scores pre- and post-training, highlighting a strong correlation between assertiveness and overall communication abilities [14]. Assertiveness training helps nurses express their thoughts and feelings appropriately in the workplace.

Mahmoudirad et al. describe assertiveness training as involving developmental factors and self-learning [23]. Developmental factors for nurses include “family training”, “social training”, and “inheritance”. The self-learning aspect encompasses conditions for learning assertive behaviors, highlighting participants’ eagerness to learn through “role modeling”, “reflective learning”, “mentoring”, “studying”, and “counseling”. However, Oducado and Montaño noted that many nurses have limited educational and training opportunities [18].

#### 3.2.4. Interaction with Other Staff

Three of the reviewed studies indicated that some nurses hesitate to be assertive due to concerns about their relationships. Omura et al. found that, while some participants acknowledged that assertive communication could foster mutual understanding, others viewed it as a risk to team harmony [26]. Oducado and Montaño noted that nurses tend to practice more affirmative assertive behaviors, such as giving compliments, encouraging opinions, making suggestions, providing constructive criticism, expressing disagreements, and resolving conflicts [18]. The literature suggests that nurses are less assertive towards nursing management personnel than their co-workers [17,18]. Newly Qualified Nurses (NQNs) tend to only challenge unsafe practices if they have strong personalities [18,20].

Mahmoudirad et al. suggest that understanding staff psychology, including analyzing moods, can improve assertive interactions among nurses [23]. For instance, if a nurse encounters a co-worker in a bad mood, knowing their psychological state allows for problem resolution rather than issuing orders. The study emphasizes the importance of mutual understanding between head nurses and assistant nurses to foster assertive behavior, noting that a lack of such understanding can lead to conflicts.

Assertiveness enhances communication and social competence, vital for attracting and retaining employees [1]. Their study found significant improvements in psychological well-being and work engagement among early career nurses after intervention programs. This development of assertive communication skills increased participants’ competence and influence, fostering better relationships that reduce stress and boost job satisfaction.

#### 3.2.5. Patient Relationship

Assertive communication with patients fosters therapeutic relationships and enhances patient care [1]. Omura et al. highlighted that nurses’ commitment to patient-centered treatment supports assertive interactions [21]. Lee et al. emphasized the importance of open communication from nurses for patient safety and assertive engagement with colleagues and physicians [22]. Additionally, a sense of responsibility, along with knowledge and confidence, facilitates assertive behavior at work.

Omura et al. found that nurses recognized the link between assertive communication and improved patient outcomes [25]. Law and Chaw illustrated this with a case where a nurse’s assertiveness in advocating for a patient prevented unnecessary suffering [24]. Moreover, assertiveness in patient relationships correlates with reduced workplace stress for nurses. Abdelaziz et al. [1] argued that assertiveness helps diminish negative emotions like anger and anxiety, especially when facing challenges such as unstable patients, staff shortages, and role conflicts.

## 4. Discussion

This systematic review explored nurses’ perspectives on assertiveness by analyzing its role, barriers, facilitators, and impact in healthcare environments. The study advances knowledge by delving into the assertiveness trait of nurses in quantitative and qualitative studies. Fifteen articles selected revealed key themes as regards to the benefit of assertiveness and factors that inhibit and enhance the assertiveness trait in nurses. The paper revealed not only the facilitators and barriers of assertiveness but also the impact of assertiveness training, assertiveness in interaction with other staff, and assertiveness in patient relationships. Similarly, Omura et al. had only eight studies that met the inclusion criteria for assertiveness training intervention [3]. This shows the limited number of literatures in assertiveness research.

This research demonstrated that personal traits like self-confidence, bravery, professionalism, and religious belief enhances assertiveness. In Lee et al., personal traits or individual factors were also found to boost assertiveness [22]. Furthermore, Morrow et al. and Okuyama et al. confirmed that personal traits like motivation, job satisfaction, and experience make nurses more likely to be assertive [27,28]. Apart from the effect of personal traits as boosters of assertiveness, certain literatures establish a link between assertiveness training and personal traits which consequently improves assertiveness. Abdelaziz et al. explored the effectiveness of assertiveness training on the personal factor of work engagement and found the training influential [1]. This reveals that assertiveness training can lead to the improvement of personal characteristics. This study also found supportive work culture to be critical in making it easier for nurses to be assertive. Similarly, Ortiz identified the importance of a supportive work environment or culture in facilitating new nurses’ assertiveness [29]. Schwappach and Richard who conducted a cross-sectional survey in Switzerland found that a work environment or culture that supports assertiveness is important for facilitating assertiveness [30].

On the other hand, barriers mostly identified in the selected articles were the fear of consequences of being assertive, hierarchy and power differentials, and negative effects on team harmony or relationship strain. Nurses’ ability to communicate effectively was hindered by fear of consequences as revealed by Reese et al. and Oducado; fear of repercussions from the nursing management personnel was the main barrier to assertiveness [31,32]. Hierarchy and power differentials were also found to be barriers to nurses’ ability to communicate assertively. Studies by Schwappach and Niederhauser and Kearns et al. demonstrated that hierarchy and power differentials made it less likely for nurses to be assertive or communicate openly in the Western part of the world [33,34]. Similarly, Krenz et al., whose study was conducted in Switzerland, found that when doctors are present, nurses were more hesitant to voice their concerns or suggestions, showing a likely power differential between nurses and doctors [35]. The negative effect on team harmony is yet another barrier to assertiveness. According to Omura et al. and Lee et al., maintaining harmonious working relationships and avoiding tensions were factors that restricted assertiveness in nurses [21,36]. Nakamura et al. and Suzuki et al. revealed that assertiveness training helped nurses in practicing assertiveness with co-workers the right way as it helped nurses to gain self-actualization without infringing on the rights of others [37,38].

The study revealed that the trainings conducted to make nurses more assertive were impactful. These trainings made the psychological well-being of nurses improve. Similarly, Parray and Kumar and Suzuki et al. in their study identified assertiveness training as impactful, as it made nurses more confident and improved nurses’ ability to communicate effectively and nurses’ well-being [38,39]. Consequently, Parray and Kumar believed that the assertiveness training led to nurses improving their care for their patient and succeeding in their careers [39]. Ayhan and Seki Öz and Hadavi and Nejad also found that assertiveness training is impactful [40,41]. They believed that it boosted the assertiveness and communication skills of nurses. According to Ibrahim and Maheshwari and Gill, assertiveness training has a positive effect on self-confidence, self-confidence, problem-solving, and decision-making processes [42,43].

The themes of assertiveness, in regards to the nurses’ relationship with other health workers, showed that nurses were less likely to be assertive with nurse managers. This is because nurses desired to avoid getting their colleagues or superiors upset. Similarly, Omura et al. found that causing disharmony and tension among teams or concerns about upsetting a colleague restrict assertiveness [21]. Apart from fears of upsetting others, the study revealed that nurses were found to be less assertive with superiors. According to Gawad et al. and Abdelaziz et al., novice nurses often struggle with assertiveness when dealing with supervisors [1,44]. The result further revealed assertiveness in patient interaction, focusing on patient safety as core.

The result mostly revealed, in terms of patient interaction, that patient safety motivates assertiveness. According to Schwappach and Niederhauser and Abdelaziz et al., nurses’ concern for patients lead them to be assertive in advocating for patients and voicing out their concerns about patient safety [1,33]. Timmins and McCabe revealed that participants cited their responsibility to patients as a primary facilitator of their assertive behavior [45]. Rainer posited that nurses are best-positioned to advocate for patients [46]. In line with this spirit of advocacy, Okuyama et al. and Schwappach and Gehring thought that perceived risk to patient safety is an important motivation for speaking up [28,47]. 

### 4.1. Limitations

This review has several limitations that should be considered when interpreting the findings. First, many included studies were conducted in collectivist societies, such as Japan and Egypt, where cultural norms and values may significantly influence assertiveness. This limits the generalizability of the findings to individualistic contexts where communication and interpersonal dynamics differ. Second, the majority of studies focused on novice nurses, potentially overlooking the perspectives and experiences of more seasoned professionals. Third, methodological heterogeneity among the included studies, such as the use of qualitative, cross-sectional, and quasi-experimental designs, restricted the feasibility of conducting a meta-analysis and may introduce variability in outcomes. Fourth, restricting the review to English-language studies may have excluded relevant research published in other languages, potentially narrowing the scope of the synthesis. Finally, sample sizes in some included studies were relatively small, limiting the robustness of certain findings. Future research should address these limitations by incorporating diverse cultural settings, a broader range of nursing experience levels, and rigorous methodologies to enhance the generalizability and applicability of the results.

### 4.2. Implications for Future Research

While the existing literature sheds light on the importance of assertiveness in nursing, there remains a gap in understanding its long-term effects on care within the field. It is imperative to conduct studies to observe the lasting impacts. Additionally, qualitative research is needed to explore nurses’ subjective viewpoints and encounters related to assertiveness in their contexts, particularly in culturally diverse settings. Further exploration is required in examining assertiveness within treatment environments. Research, in this area, could provide insights for customizing assertiveness training initiatives based on environmental factors. The influence of culture on assertiveness within nursing contexts remains an unexplored area presenting an opportunity for future investigation.

## 5. Conclusions

Assertiveness is crucial for nurses to voice their opinions and concerns as advocates, fostering communication and teamwork among colleagues. It plays a significant role in establishing harmonious working relationships within healthcare settings. Assertiveness, centered on self-respect and communication abilities, helps nurses achieve professional growth while respecting others’ rights.

Moreover, assertiveness enhances nurse–patient communication, leading to better patient education, clearer explanations of care plans, and increased patient trust. Studies indicate that assertiveness training improves nurses’ ability to handle conflicts, reduce workplace stress, and enhance job satisfaction, ultimately leading to better patient outcomes and safer healthcare environments. Prior research indicates that enhancing assertiveness skills correlates with increased levels of confidence, self-efficacy, and professional autonomy, allowing nurses to be more effective in their roles while improving interdisciplinary collaboration.

## Figures and Tables

**Table 1:** SPIDER format.

Elements of SPIDER	Elements of SPIDER as Applied to This Review
S—Sample	Registered nurses.
PI—Phenomenon of Interest	Assertiveness.
D—Design	Studies using qualitative or quantitative designs.
E—Evaluation	Nurses’ experiences, perceptions, and opinions; views related to assertiveness.
R—Research type	Primary research sources of both qualitative and quantitative research designs.

**Table 2:** Study characteristics.

Author	Year	Country	Aim/Objective	Sample Work Environment	Design	Tools	Results	Conclusions
Abdelaziz et al. [1]	2020	Egypt	Effectiveness of an assertiveness training program on psychological well-being and work engagement among novice psychiatric nurses.	36 novice psychiatric nurses at The Abbasia hospital for mental health.	Quasi-experimental design.	Socio-demographic data sheet, Rathus Assertiveness Schedule, Riff’s Psychological Well-Being Scale, and Utrecht Work Engagement Scale.	Statistically significant improvement in assertiveness skills, psychological well-being, and work engagement. Positive correlation between assertiveness skills and psychological well-being.	Assertiveness training improves well-being and engagement in novice nurses, suggesting benefit in structured training programs for skills development.
Mostafa et al., [14]	2022	Egypt	Assess the effect of an assertiveness training program on communication skills and self-esteem among psychiatric nurses.	50 nurses at the Psychiatric Mental Health Hospital.	Quasi-experimental design.	I: Structured interview questionnaire. II: Rosenberg’s Global Self-esteem Scale. III: Assertiveness skills scale. IV: Communication Skills Inventory.	Significant improvement in self-esteem, assertiveness, and communication skills post-training.	Training enhanced essential interpersonal skills, promoting self-confidence and patient care quality.
Mohammed et al., [15]	2022	Egypt	Assess communication skills training impact on head nurses’ assertiveness and self-esteem.	50 head nurses at Nasser Institute Hospital for Research and Treatment.	Quasi-experimental design.	The data of this study were collected through three tools, namely, a knowledge questionnaire, an Assertiveness Assessment Scale, and a Sorensen Self-Esteem Scale.	Improved knowledge, assertiveness, and self-esteem post-intervention. Assertiveness increased from 70% to 94%; self-esteem increased from 34% to 78% (*p* < 0.001).	Communication training effectively boosted assertiveness and self-esteem.
Khanam et al. [16]	2023	Pakistan	Evaluate effectiveness of assertiveness training on psychological well-being and work engagement among novice nurses.	36 novice nurses working in a psychiatric department.	Quasi-experimental design.	Assertiveness, psychological well-being, and work-engagement was collected using three adopted questionnaires.	Significant increase in psychological well-being (132.14 to 188.06), assertiveness (8.94 to 34.56), and work engagement (65.17 to 78.39).	Training supports psychological well-being and engagement, enhancing adaptability and resilience for novice nurses.
Nemati et al., [13]	2021	Iran	Evaluate impact of assertiveness training program on assertiveness and self-esteem among nurses.	70 nurses at Imam Reza Hospital.	Experimental design.	The Coopersmith Self-Esteem Questionnaire, the Omali and Bachman Self-Esteem Scale, the Rosenberg Self-Esteem Scale, and the self-reporting questionnaire.	Intervention group showed increased assertiveness and self-esteem post-intervention, but not significant compared to control group.	Training led to improvement, but long-term or more intensive programs may be needed to achieve lasting results.
Oducado and Montaño [18]	2021	Philippines	Assess workplace assertiveness among hospital staff nurses toward colleagues, management, and other health team members.	223 nurses at two tertiary hospitals.	Cross-sectional design.	Workplace assertive behavior questionnaire. Descriptive statistics and tests for differences were used to analyze the data.	Moderate assertiveness in workplace. Assertiveness varied by employment status, age, experience, and organizational tenure.	Assertiveness influenced by workplace hierarchy and norms, indicating need for management support to foster assertive communication.
Wehabe et al., [17]	2018	Egypt	Investigate relationship between assertiveness and leadership styles among head nurses.	98 head nurses at Ain-Shams University Hospitals.	Analytic cross-sectional design.	A self-administered questionnaire which included two different tools, assertiveness scale and the Multifactor Leadership Questionnaire (MLQ).	A total of 77.6% had high assertiveness; assertiveness correlated with transformational and transactional leadership styles and negatively correlated with passive/avoidant styles.	Assertiveness positively linked with proactive leadership, suggesting assertiveness training could enhance leadership effectiveness in healthcare.
Marahatta & Koirala, [19]	2022	Nepal	The objective of the study was to find out the level of assertiveness and self-esteem.	155 nurses at Chitwan Medical College, Teaching Hospital.	Descriptive cross-sectional design.	Self-administered structured questionnaire consisted of three parts. First part consists of socio-demographic and professional information, second part consists of Simple Rathus Assertiveness Schedule (SRAS), and third part consists of Rosenberg Self-Esteem Scale (RSES).	A total of 51% high assertiveness, 54.8% high self-esteem. Assertiveness and self-esteem associated with age, ethnicity, residence, marital status, education, and job satisfaction.	Many nurses show low assertiveness and self-esteem, highlighting a need for training to boost confidence and patient care.
Mansour, M., & Mattukoyya, R. [20]	2019	East England	To examine newly qualified nurses’ views on how nursing preceptorship programs contribute to shaping their assertive communication skills.	42 nurses from four acute hospital trusts in east England.	Cross-sectional design.	Open-ended questions included in a cross-sectional survey that was analyzed using thematic analysis.	Themes included enthusiasm vs. skepticism, supportive work culture, and logistical challenges.	Preceptorship programs support assertive communication skills. Ongoing support and organizational commitment needed.
Omura et al. [21]	2018	Japan	Explore nurses’ perceptions of assertive communication in Japanese healthcare and identify factors impacting assertiveness.	23 nurses at workplaces or universities.	A belief elicitation qualitative study informed by the Theory of Planned Behavior.	Individual face-to-face semi-structured interviews.	Hierarchies, age-based seniority, and fear of offending colleagues hindered assertive communication. Novice nurses reluctant to speak up.	Hierarchical and cultural barriers affect assertive communication, suggesting need for culturally adapted assertiveness training.
Lee et al., [22]	2022	Korea	Identify factors motivating or inhibiting nurses’ speaking-up behaviors.	15 nurses from four Korean hospitals.	Descriptive qualitative design.	Semi-structured interviews.	Speaking up motivated by safety culture, supportive managers, and role models and inhibited by hierarchies, seniority, and heavy workload.	Cultural and organizational factors significantly influence speaking-up behavior, underlining the need for a supportive work culture to encourage open communication for patient safety.
Mahmoudirad et al., [23]	2009	Iran	Explore the assertiveness process among Iranian nursing leaders.	12 nurse managers working in four hospitals in Iran.	Grounded theory qualitative design.	Semi-structured interviews.	Assertiveness influenced by external/internal factors and shaped significantly by religious beliefs.	Assertiveness in Iranian nurse leaders is shaped by cultural and religious values, suggesting the need for training that respects these influences.
Law & Chan, [24]	2015	Hong Kong	Explore the learning process of speaking up among newly graduated nurses.	18 newly graduated nurses from seven public hospitals in Hong Kong.	Narrative qualitative design.	Unstructured interviews and emails.	Identified need for ongoing mentoring and creation of safe communication environments as critical for empowering nurses to speak up.	New nurses benefit from mentoring and supportive environments for developing assertive communication, particularly in hierarchical cultures like Hong Kong.
Omura, Stone, & Levett-Jones [25]	2018	Japan	Explore cultural influences on Japanese nurses’ assertive communication.	23 registered nurses in hospitals, communities, and educational institutions.	Descriptive qualitative design.	Face-to-face interviews with a semi-structured format.	Cultural values of “wa” (harmony), collectivism, and hierarchy inhibit assertive communication. Speaking up perceived as disruptive to team harmony.	Training programs should be culturally adapted, addressing barriers like collectivism and hierarchy to support assertive communication in Japanese healthcare settings.
Omura, Stone, & Levett-Jones [26]	2018b	Japan	Investigate hierarchy and power’s impact on Japanese nurses’ assertive communication.	23 registered nurses in hospitals, communities, and educational institutions.	Phenomenological qualitative design.	Face-to-face interviews with a semi-structured format.	Identified hierarchy, professional status, seniority, gender imbalance, and cultural humility as barriers to assertive communication.	Assertiveness training must account for hierarchical and cultural values, emphasizing indirect communication strategies for effective adaptation in Japanese healthcare.

**Table 3:** Thematic mapping.

	Study	Training	Barriers	Facilitators	Patient Relationships	Interaction with Other Staff
1	Abdulaziz et al. [1]	X				X
2	Mostafa et al., [14]	X		X	X	X
3	Mansour and Mattukoyya [20]	X	X	X	X	X
4	Marahatta and Koirala [19]	X	X	X		
5	Wehabe et al., [17]	X		X	X	X
6	Mohammed et al. [15]	X		X		X
7	Nemati et al. [13]	X		X	X	X
8	Oducado and Montaño [18]	X	X	X	X	X
9	Khanam et al. [16]	X		X	X	X
10	Omura et al. [21]	X	X	X	X	X
11	Lee et al., [22]		X	X		X
12	Law & Chan [24]	X	X	X		X
13	Mahmoudirad et al., [23]	X	X	X		X
14	Omura, Stone, & Levett-Jones [25]	X	X			X
15	Omura, Stone, & Levett-Jones [26]	X	X			X

**Table 4:** Themes and subthemes.

Theme	Subthemes
Facilitators of assertiveness	Age and level of qualification; Personal characteristics and beliefs; Responsibility to patients; Supportive environment; Assertiveness training program; Positive assertive experience; Safety culture; Work experience.
Barriers to assertiveness	Assertiveness is termed as being impolite; Fear of consequences of being assertive; Hierarchy and power differentials; Skills gap; Culture of maintaining group harmony; Heavy workload; Lack of support and role models.
Assertiveness training	Development of assertiveness skill; Improves psychological well-being of nurses; Professionalism; Training answers self-learning; Training is based on the understanding that nurses are humans with value; Effective communication; Limited assertiveness training; Self-esteem; Training improves self-confidence and self-actualization.
Interaction with other staff	Attraction and retention of work relationships through communication skills; Assertiveness reduces stress and increases job satisfaction; Assertiveness is beneficial; Less assertive with nursing managers; Refraining from being assertive based on concerns for relationship; Exhibited moderate assertiveness with other nurses; Method of assertiveness; Understanding staff mood.
Patient relationship	Concern for a patient as a primary motivation; Patient outcomes; Minimizes workplace stressors; Therapeutic relationship and quality care.

## Data Availability

No new data were created or analyzed in this study.

## Public Involvement Statement

No public involvement in any aspect of this research.

## Guidelines and Standards Statement

This manuscript was drafted against the PRISMA (Preferred Reporting Items for Systematic Reviews and Meta-Analyses) guidelines for systematic reviews and meta-analyses (Page et al., 2020) [7].

## Abbreviations

The following abbreviations are used in this manuscript:

JBI	Joanna Briggs Institute
CASP	Critical Appraisal Skills Program
PRISMA	Preferred Reporting Items for Systematic Reviews and Meta-Analyses
SPIDER	Sample, Phenomenon of Interest, Design, Evaluation, Research type

## Appendix A

It includes the following:

1. Summary of the quality using JBI and CASP checklist;
2. Risk of bias summary according to each domain.

**Table A1:** CASP items.

CASP Items	Mansour & Mattukoyya [20]	Omura et al. [21]	Lee et al. [22]	Law & Chan [24]	Mahmoudirad et al. [23]	Omura, Stone, & Levett-Jones [25]	Omura, Stone, & Levett-Jones [26]
Was there a clear statement of the aims of the research?	2	2	2	2	2	2	2
Is a qualitative methodology appropriate?	2	2	2	2	2	2	2
Was the research design appropriate to address the aims of the research?	2	2	2	2	2	2	2
Was the recruitment strategy appropriate to the aims of the research?	2	2	2	2	2	2	2
Was the data collected in a way that addressed the research issue?	2	2	2	2	2	2	2
Has the relationship between researcher and participants been adequately considered?	2	2	2	2	2	2	2
Have ethical issues been taken into consideration?	2	2	2	2	2	2	2
Was the data analysis sufficiently rigorous?	2	2	2	2	2	2	2
Is there a clear statement of findings?	2	2	2	2	2	2	2
How valuable is the research?	2	2	2	2	2	2	2
Total score	20	20	20	20	20	20	20

**Table A2:** JBI items: quasi-experimental studies.

JBI Items Quasi-Experimental Studies	Abdelaziz et al. [1]	Mostafa et al. [14]	Mohammed et al. [15]	Khanam et al. [16]	Nemati et al. [13]
Is it clear in the study what is the ‘cause’ and what is the ‘effect’ (i.e., there is no confusion about which variable comes first)?	3	3	3	3	3
Were the participants included in any comparisons similar?	3	3	0	0	3
Were the participants included in any comparisons receiving similar treatment/care, other than the exposure or intervention of interest?	3	3	0	3	0
Was there a control group?	3	2	2	2	3
Were there multiple measurements of the outcome both pre and post the intervention/exposure?	3	3	3	3	3
Was follow up complete and if not, were differences between groups in terms of their follow up adequately described and analyzed?	3	0	3	3	3
Were the outcomes of participants included in any comparisons measured in the same way?	3	3	3	3	3
Were outcomes measured in a reliable way?	3	3	3	3	3
Was appropriate statistical analysis used?	3	3	3	3	3
Total Score	27	23	20	23	24

**Table A3:** JBI items: analytical cross-sectional studies.

JBI Items Analytical Cross-Sectional Studies	Marahatta & Koirala [19]	Wehabe et al. [17]	Oducado & Montaño [18]
Were the criteria for inclusion in the sample clearly defined?	3	3	3
Were the study subjects and the setting described in detail?	3	3	3
Was the exposure measured in a valid and reliable way?	3	0	2
Were objective, standard criteria used for measurement of the condition?	3	2	2
Were confounding factors identified?	3	0	0
Were strategies to deal with confounding factors stated?	1	3	3
Were the outcomes measured in a valid and reliable way?	3	3	3
Was appropriate statistical analysis used?	3	3	3
Total Score	22	17	19
